# Prognostic impact of micropapillary component in patients with node‐negative subcentimeter lung adenocarcinoma: A Chinese cohort study

**DOI:** 10.1111/1759-7714.13702

**Published:** 2020-10-15

**Authors:** Jie Yao, Erjia Zhu, Ming Li, Jinshi Liu, Lei Zhang, Honggang Ke, Hang Su, Huikang Xie, Guanxin Xu, Ling Zhu, Junqiang Fan, Chang Chen, Surgical Thoracic Alliance of Rising Star Collaborative Group

**Affiliations:** ^1^ Department of Thoracic Surgery The Second Affiliated Hospital of Zhejiang University School of Medicine Hangzhou China; ^2^ Department of Thoracic Surgery Shanghai Pulmonary Hospital, Tongji University School of Medicine Shanghai China; ^3^ Department of Thoracic Surgery, Jiangsu Cancer Hospital Nanjing Medical University Nanjing China; ^4^ Department of Thoracic Surgery Zhejiang Cancer Hospital Hangzhou China; ^5^ Department of Thoracic Surgery The First People's Hospital of Changzhou Changzhou China; ^6^ Department of Thoracic Surgery Affiliated Hospital of Nantong University Nantong China; ^7^ Department of Pathology, Shanghai Pulmonary Hospital Tongji University School of Medicine Shanghai China

**Keywords:** Micropapillary component, subcentimeter lung adenocarcinoma

## Abstract

**Background:**

In this study, we investigated the prognostic significance of a micropapillary (MP) component in patients with subcentimeter lung adenocarcinoma.

**Methods:**

A total of 311 patients with subcentimeter lung adenocarcinoma who underwent surgical resection between January 2009 to December 2012 from seven medical centers were included. Recurrence‐free survival (RFS) and overall survival (OS) were analyzed.

**Results:**

The five‐year RFS was 79.8% in 97 (97/311, 31%) cases of adenocarcinoma with a MP component and 93.5% in the 214 (214/311, 69%) cases without. In multivariate analysis, MP was an independent risk factor for worse RFS (hazard ratio [HR], 3.73; 95% confidence interval [CI]: 1.87–7.42; *P* < 0.001) and OS (HR, 5.84; 95% CI: 2.20–15.49; *P* < 0.001). There was no significant difference among wedge resection, segmentectomy and lobectomy on RFS (*P* = 0.256) and OS (*P* = 0.103) in patients without MP. Regarding patients with a MP component, lobectomy achieved equivalent prognosis than segmentectomy, and both were better than wedge resection (*P* = 0.001).

**Conclusions:**

A MP component still suggest a poor prognosis in subcentimeter lung adenocarcinoma. Patients with subcentimeter lung adenocarcinoma with a MP component of 5% or greater treated with wedge resection were at higher risk of recurrence than patients treated with anatomical resection.

## Graphical abstract

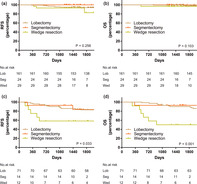

Presence of a micropapillary component in subcentimeter lung cancer indicated that anatomical resection should be performed instead of wedge resection.

## Introduction

Lung cancer is now the leading cause of cancer‐related death in the world and lung adenocarcinoma has become the most common histological type.^1^ In 2011, the International Association for the Study of Lung Cancer (IASLC)/American Thoracic Society (ATS)/and European Respiratory Society (ERS) proposed a new classification and recommended that lung adenocarcinoma should be classified into five subtypes according to their predominant histological component.^2^ The prognostic impact of micropapillary (MP) and/or solid predominant subtype on survival outcomes in lung adenocarcinoma has been demonstrated to be associated with worse prognosis.^3^, ^4^, ^5^, ^6^ However, the clinicopathological characteristics and long‐term survival in patients with adenocarcinoma harboring a minor proportion of MP component (nonpredominant) remains unclear.

With the development of radiological techniques in high‐resolution computed tomography (HRCT) scanning, the detection rate of small‐sized nodules, especially subcentimeter nodules (tumor size ≤1 cm), has gradually increased.^7^ Several studies have reported that subcentimeter lung adenocarcinoma is associated with lower rate of nodal metastasis and recurrence, with a better long‐term patient survival.^8^, ^9^ Sublobar resection in these patients including segmentectomy and wedge resection has been reported to have an equivalent prognosis compared with lobectomy.^10^, ^11^, ^12^ However, the National Comprehensive Cancer Network (NCCN) guidelines propose lobectomy and systematic lymph node dissection as the standard procedure for early‐stage lung adenocarcinoma.^13^ In addition, it has been recommended that lobectomy is the optimal choice to provide a potential cure in patients with small‐sized lung adenocarcinoma MP predominant subtype.^14^ However, lung adenocarcinoma often harbors multiple histological subtypes.^15^ To date, whether patients with MP component (predominant or nonpredominant) in subcentimeter lung adenocarcinoma may benefit from less intensive resection has not been investigated.

In this study, we aimed to evaluate the prognostic value and appropriate surgical procedure of MP subtype in subcentimeter lung adenocarcinoma from a multicenter study group.

## Methods

### Patient population

This retrospective study was approved by the Institutional Review Board of The Second Affiliated Hospital of Zhejiang University, Shanghai Pulmonary Hospital, Zhejiang Cancer Hospital, Jiangsu Cancer Hospital, Affiliated Hospital of Nantong University and The First People's Hospital of Changzhou, on behalf of the Surgical Thoracic Alliance of Rising star group (STAR). Patients with surgically resected c‐N0 subcentimeter lung cancer who underwent curative resection from seven medical centers between January 2009 and December 2012 were included in the study. The inclusion criteria were (i) tumor size ≤1 cm; (ii) nonenlarged lymph nodes on CT scan; and (iii) pathologically diagnosed lung adenocarcinoma. The exclusion criteria were as follows: (i) previous neoadjuvant chemotherapy; (ii) presence of multiple primary cancer; (iii) incomplete follow‐up information; and (iv) positive surgical margin. Ultimately, a total of 311 patients with subcentimeter lung adenocarcinoma were included in our study. Patients' demographic and clinical data, disease extent, treatment and follow‐up information were collected.

### Preoperative evaluation

Preoperative staging was performed with chest CT scan, brain MRI, and bone scintigraphy. Hilar or mediastinal lymph nodes larger than 1 cm in the short axis on CT images were defined as clinical N factor positive. Preoperative positron‐emission tomography (PET) scan was performed at the discretion of the individual clinician, especially in patients with suspicion of lymph node metastasis. Mediastinoscopy was not performed unless suspicious lymphadenopathy was indicated on CT or PET scan. Surgical resection was indicated for patients without pathological evidence of mediastinal lymph node involvement including those with hilar nodal disease on CT or PET scan.

### Surgical indication and tumor margin

Lobectomy with systematic hilar and mediastinal lymph node dissection was the primary procedure. The indication for limited resection was decided on the basis of a combination of the performance status of patients and CT findings. Sublobar resections, including anatomical segmentectomy and wedge resection, were indicated for patients with peripheral lesions located at the outer one‐half of lung parenchyma, or patients with shadows composed mainly of ground‐glass nodules on CT. In contrast, we performed compromised limited resection for high‐risk patients with any contraindications for standard radical surgery, regardless of the tumor size or presence of a solid component on CT. The surgical technique was aimed at securing sufficient margins of at least 2.0 cm. The tumor margin was defined as the distance from the primary tumor to the closest staple lines. The tumor margin distance was measured by pathologists, and the tumor margin distances recorded the pathological reports were used for the analyses.

### Histopathological evaluation

**Figure 1:**
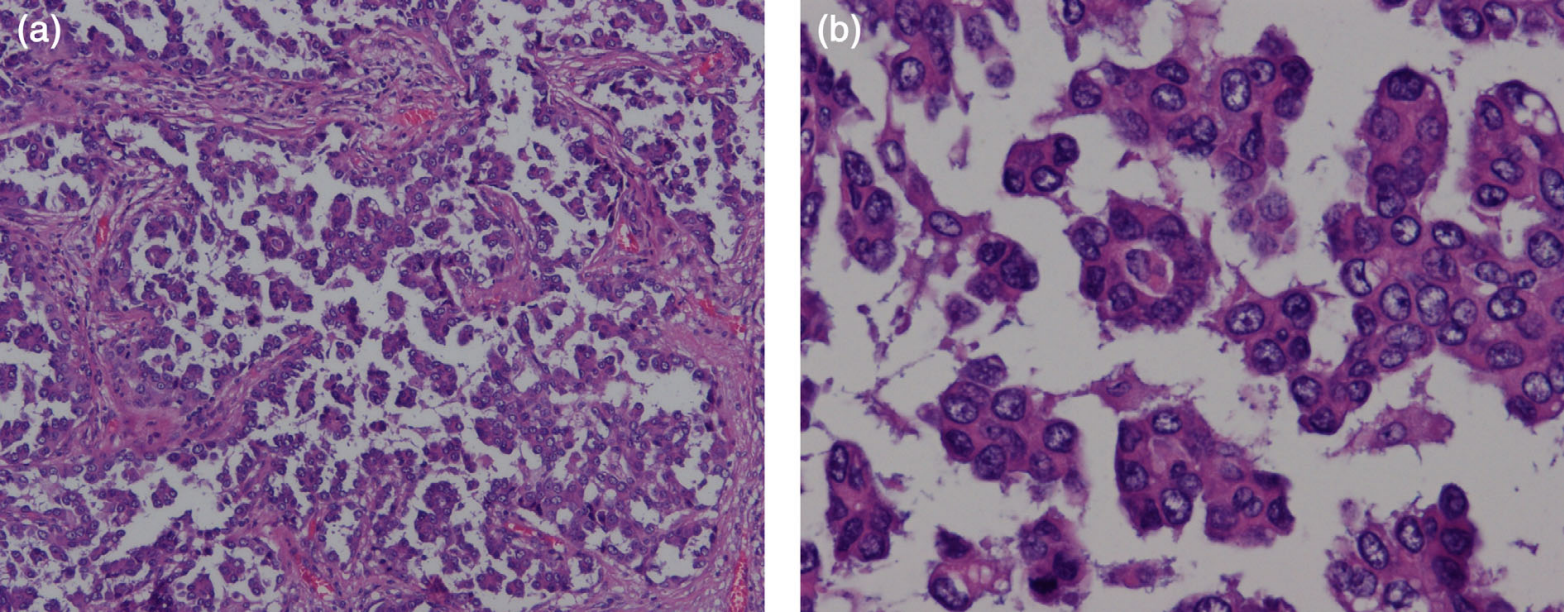
Micropapillary adenocarcinoma consists of small papillary clusters of glandular cells growing within the airspace which lack fibrovascular cores (a, ×100; b × 200).

All patients' pathological slices were reviewed and reclassified by two clinical pathologists in each center who were blinded to patient characteristics and clinical information. When disagreement occurred, a third senior pathologist was enlisted in order to reach an agreement. According to the 2015 WHO classification of lung adenocarcinoma, invasive lung adenocarcinoma was divided into lepidic, acinar, papillary, solid, and MP subtypes based on the predominant histological pattern presenting in the tumor. The percentage of each histological subtype was recorded in 5% increments, and a subtype was considered present if ≥5% in the tumor. The pattern with largest percentage was defined as predominant pattern of histological subtype. The micropapillary pattern was defined as (i) a floret pattern with tumor cells growing in papillary tufts forming florets that lack fibrovascular cores; and a (ii) stromal pattern with tumor cells invading stromal spaces encased by connective tissues. Representative images were added to the results section (Fig 1). Adenocarcinomas with a proportion of MP component >0% were defined as MP positive adenocarcinomas. Adenocarcinomas without any MP component were defined as MP negative adenocarcinomas.

We also retrospectively investigated the diagnostic accuracy of presence of micropapillary component by frozen section. In our cohort, all specimens were intraoperatively diagnosed by frozen section results. Two senior pathologists reported the presence of a micropapillary component using a multihead microscope and discussed the results until a consensus was achieved.

### Follow‐up policy

All patients received a physical examination, blood examination including tumor markers and chest HRCT scan every 6 to 12 months during the first two years after operation and yearly thereafter. The last follow‐up date was October 2018, the postoperative local recurrence or distant metastasis was diagnosed using chest HRCT, brain magnetic resonance imaging (MRI), and bone scanning as well as ultrasound and/or abdominal CT. The postoperative recurrence pattern was divided into locoregional, and distant recurrence. Locoregional recurrence was defined as any recurrent disease within the ipsilateral hemithorax, lung metastasis, and/or hilar or mediastinal lymph node metastasis. Distant recurrence was considered distant metastases. Recurrence was confirmed by radiological findings, including CT and/or positron emission tomography findings.

### Statistical analysis

Categorical variables were compared using the χ2 test and Fisher's exact test as appropriate, and continuous variables were compared using independent *t*‐test. Overall survival (OS) and recurrence‐free survival (RFS) were analyzed by the Kaplan‐Meier method. In addition, univariate and multivariate Cox proportional regression analysis were used to evaluate the prognostic impact of various factors. Univariate and multivariate logistic regression model was performed to determine the relationship of micropapillary component and other clinicopathological factors. The input variables in the multivariate model were those with *P*‐values less than 0.1 in the univariate analysis. A two‐sided *P*‐value of less than 0.05 indicated statistical significance. All statistical analysis was performed using SPSS version 20.0 (IBM Corporation, Armonk, NY, USA).

## Results

The overall characteristics of the patients were compared according to the positive or negative MP adenocarcinomas (Table 1). In patients with subcentimeter lung adenocarcinoma, there were 97 cases (31%) identified with a MP component, Average value of micropapillary proportion was 18.14. Median value of micropapillary proportion was 15.00. Micropapillary proportion ranged from 5.00 to 70.00. and 214 cases (69%) were identified without MP component. Gender, age, smoking history, tumor location, total size, visceral pleural invasion (VPI) and surgical procedure were not related to the presence of MP subtype, while predominant subtype had a remarkable correlation with MP presence (*P* < 0.001). Additionally, patients with lepidic‐predominant tumors had a lower probability of harboring a MP component (*P* < 0.001). The relationship between the MP positive adenocarcinomas and patient characteristics is shown in Table 2. A multivariate logistic analysis demonstrated that the presence of lepidic subtype was a significant lower risk factor in the presence of MP subtype (odds ratio [OR], 0.21; 95% confidence interval [CI]: 0.11–0.39), while gender, age, smoking history, tumor location, total size, VPI were not related to the presence of MP subtype.

**Table 1:** Patient characteristics according to the presence of a micropapillary component

Characteristics	Micropapillary (−) *N* = 214	Micropapillary (+) *N* = 97	*P*‐value
Age			0.313
≤65	168 (79)	71 (73)	
>65	46 (21)	26 (27)	
Sex			0.212
Male	80 (37)	44 (45)	
Female	134 (63)	53 (55)	
Smoking			0.881
Yes	44 (21)	21 (22)	
No	170 (79)	76 (78)	
Tumor location			0.312
Upper and middle	139 (65)	57 (59)	
Lower	75 (35)	40 (41)	
Total size (cm)			0.999
≤0.5	15 (7)	7 (7)	
>0.5	199 (93)	90 (93)	
Predominant component			**<0.001**
Lepidic	106 (50)	17 (18)	
Acinar	68 (32)	45 (46)	
Papillary	30 (14)	26 (27)	
Micropapillary	0 (0)	4 (4)	
Solid	10 (5)	5 (5)	
Lepidic pattern			**<0.001**
Present	168 (79)	37 (38)	
Absent	46 (21)	60 (62)	
Acinar pattern			0.113
Present	141 (66)	73 (75)	
Absent	73 (34)	24 (25)	
Papillary pattern			0.999
Present	139 (65)	63 (65)	
Absent	75 (35)	34 (35)	
Solid pattern			**0.032**
Present	14 (7)	14 (14)	
Absent	200 (93)	83 (86)	
VPI			0.361
Present	40 (19)	23 (24)	
Absent	174 (81)	74 (76)	
Nodal involvement			0.186
N0	212 (98)	94 (97)	
N1	1 (1)	2 (2)	
N2	1 (1)	1 (1)	
Surgery			0.714
Lobectomy	161 (75)	71 (73)	
Segmentectomy	24 (11)	14 (14)	
Wedge resection	29 (14)	12 (12)	

**Table 2:** Relationship between the presence of a micropapillary component and patient characteristics

Variables	Univariate		Multivariate	
	OR (95% CI)	*P*‐value	OR (95% CI)	*P*‐value
Sex (female)	0.72 (0.44–1.17)	0.184		
Age (>65)	1.34 (0.77–2.33)	0.305		
Smoking (yes)	1.07 (0.59–1.92)	0.827		
Tumor location (lower)	1.27 (0.73–2.22)	0.392		
Total size (cm)	1.18 (0.42–3.35)	0.752		
Predominant component				
Lepidic (reference)		**< 0.001**	^a^	
Acinar	4.13 (2.19–7.79)	**< 0.001**	^a^	
Papillary	5.40 (2.60–11.25)	**<0.001**	^a^	
Solid	3.12 (0.95–10.24)	0.061	^a^	
Lepidic (present)	0.17 (0.10–0.29)	**<0.001**	0.16 (0.09–0.28)	**<0.001**
Acinar (present)	1.58 (0.92–2.71)	0.100		
Papillary (present)	1.00 (0.61–1.65)	0.999		
Solid (present)	2.41 (1.10–5.27)	**0.028**	0.81 (0.34–1.93)	0.634
VPI (present)	1.35 (0.76–2.42)	0.309		
Nodal involvement (present)	3.38 (0.56–20.58)	0.186		

^a^ Predominant component was not included in multivariate analysis because of the association with presence of each components in adenocarcinoma. CI, confidence interval; OR, odds ratio.

**Figure 2:**
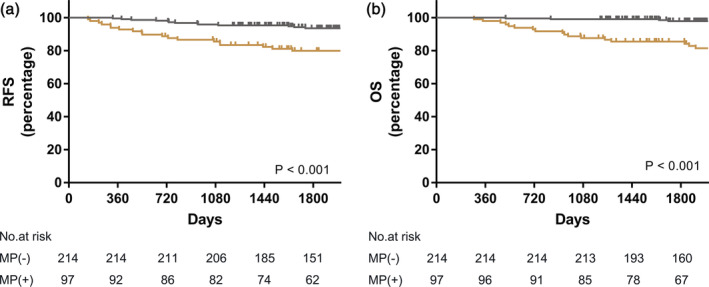
Prognostic impact on (**a**) RFS; (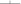) MP (−), and (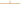) MP (+) and (**b**) OS between patients with positive or negative MP component (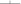) MP (−), and (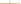) MP (+). (OS: overall survival; RFS: recurrence‐free survival; MP: micropapillary).

Patients were subclassified into MP positive and MP negative groups. The MP positive group had a significant worse OS (*P* < 0.001) and RFS (*P* < 0.001) than the MP negative group (Fig 2). In the multivariate analysis, the Cox regression model was used to adjust for gender, age, smoking history, tumor location, and total size. The five‐year OS and RFS were significantly worse for patients with MP presence (OS: HR, 5.84; 95% CI: 2.20–15.49, *P* < 0.001; RFS: HR, 3.73; 95% CI: 1.87–7.42, *P* < 0.001) (Table 3).

**Table 3:** Cox regression model to predict RFS and OS of c‐N0 subcentimeter lung adenocarcinoma

	Univariate		Multivariate	
Variables	HR (95% CI)	*P*‐value	HR (95% CI)	*P*‐value
RFS				
Sex (female)	0.48 (0.25–0.94)	**0.031**	0.53 (0.27–1.06)	0.072
Age (>65)	1.04 (0.47–2.28)	0.930		
Smoking (yes)	1.37 (0.64–2.93)	0.413		
Tumor location (lower)	0.61 (0.26–1.46)	0.266		
Total size (cm)	1.46 (0.32–6.58)	0.625		
Predominant component				
Lepidic	Reference	**0.053**	^a^	
Acinar	2.06 (0.91–4.66)	**0.083**	^a^	
Papillary	1.80 (0.67–4.84)	0.242	^a^	
Solid	0.94 (0.12–7.45)	0.956	^a^	
Micropapillary	9.52 (2.05–44.17)	**0.004**	^a^	
Lepidic (present)	0.65 (0.34–1.28)	0.214		
Acinar (present)	1.36 (0.64–2.91)	0.425		
Papillary (present)	0.60 (0.31–1.17)	0.131		
Solid (present)	1.82 (0.71–4.70)	0.214		
Micropapillary (present)	3.93 (1.98–7.82)	**<0.001**	3.90 (1.93–7.87)	**<0.001**
VPI (present)	0.69 (0.23–2.03)	0.495		
Nodal involvement (present)	6.83 (2.09–22.33)	**0.001**	5.42 (1.58–18.65)	**0.007**
Surgery				
Lobectomy	reference	**0.051**		**0.009**
Segmentectomy	0.61 (0.14–2.57)	0.496	0.45 (0.10–1.94)	0.283
Wedge resection	2.50 (1.11–5.60)	**0.026**	3.13 (1.38–7.13)	**0.007**
OS				
Sex (female)	0.58 (0.27–1.28)	0.179		
Age (> 65)	1.77 (0.76–4.12)	0.184		
Smoking (yes)	1.66 (0.69–4.01)	0.257		
Tumor location (lower)	0.78 (0.30–2.06)	0.616		
Total size (cm)	1.66 (0.26–10.39)	0.590		
Predominant component				
Lepidic (reference)		**0.001**	a	
Acinar	1.95 (0.71–5.38)	0.195	a	
Papillary	1.97 (0.60–6.45)	0.264	a	
Solid	1.43 (0.17–11.93)	0.738	a	
Micropapillary	19.76 (4.85–80.42)	**<0.001**	a	
Lepidic (present)	0.46 (0.21–1.00)	**0.050**	0.91 (0.35–2.39)	0.849
Acinar (present)	1.24 (0.52–2.98)	0.626		
Papillary (present)	0.58 (0.26–1.26)	0.169		
Solid (present)	2.58 (0.97–6.88)	**0.059**	1.49 (0.48–4.61)	0.489
Micropapillary (present)	5.73 (2.39–13.76)	**<0.001**	6.09 (2.33–15.94)	**<0.001**
VPI (present)	0.18 (0.02–1.35)	**0.095**	0.19 (0.02–1.41)	0.104
Nodal involvement (present)	4.82 (1.11–20.93)	**0.036**	4.50 (1.01–20.00)	**0.048**
Surgery				
Lobectomy (reference)		**0.002**		**0.002**
Segmentectomy	0.62 (0.08–4.80)	0.651	0.47 (0.06–3.69)	0.470
Wedge resection	4.61 (1.92–11.08)	**0.001**	4.61 (1.86–11.46)	**0.001**

^a^ Predominant component was not included in multivariate analysis because of the association with presence of each components in adenocarcinoma. CI, confidence interval; HR, hazard ratio.

**Figure 3:**
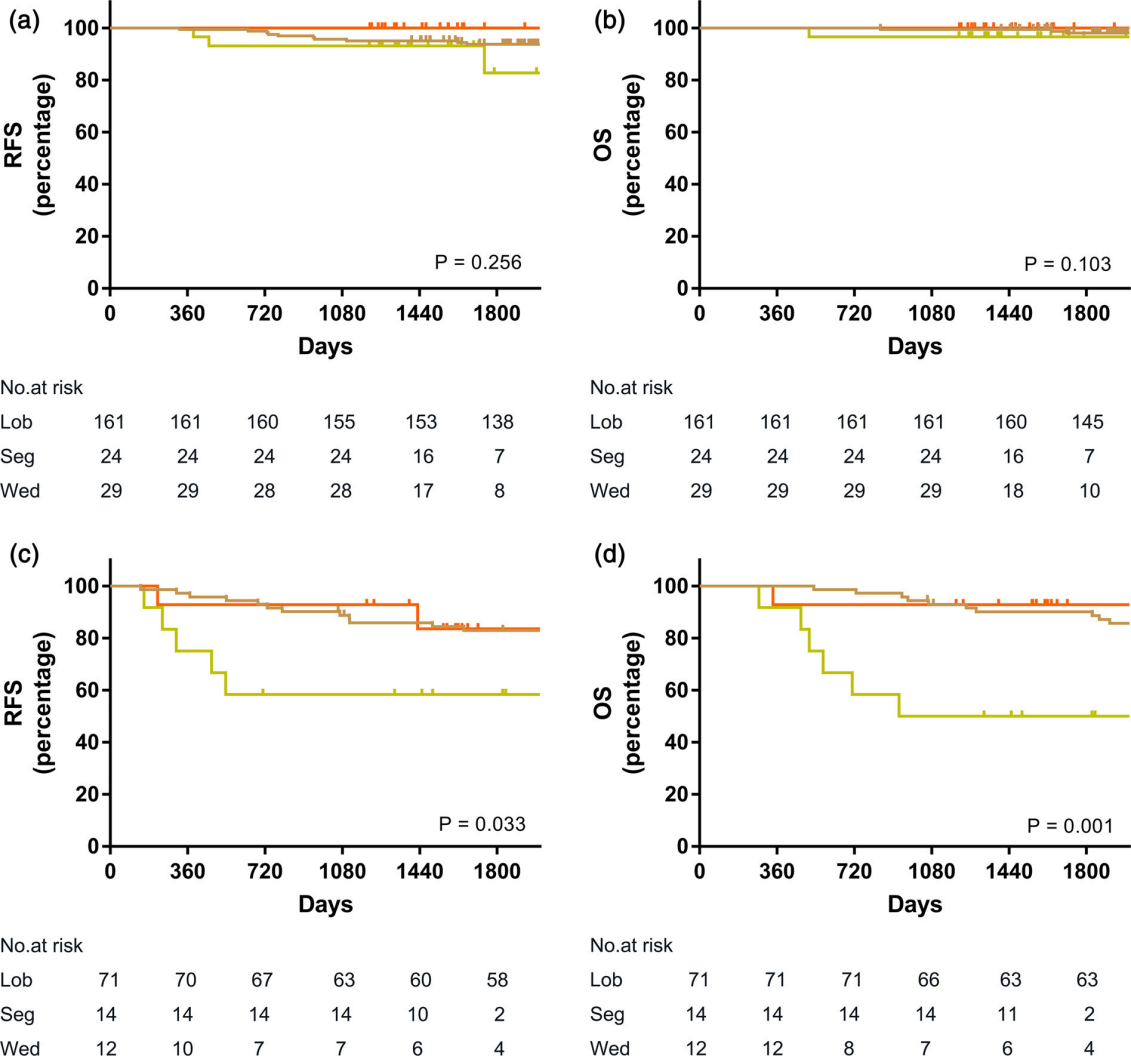
(**a**, **b**) Prognostic impact of surgical procedure on RFS and OS in patients with negative MP component; (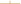) Lobectomy, (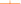) Segmentectomy, and (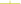) Wedge resection; (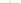) Lobectomy, (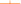) Segmentectomy, and (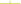) Wedge resection or (**c**, **d**) positive MP component (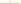) Lobectomy, (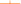) Segmentectomy, and (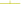) Wedge resection; (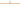) Lobectomy, (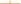) Segmentectomy, and (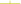) Wedge resection. (OS: overall survival; RFS: recurrence‐free survival; MP: micropapillary; Lob, lobectomy; Seg, segmentectomy; Wed, wedge resection).

For all 79 patients who underwent limited resection, no patients had a margin distance ≤10 mm, 47 (60%) patients had a margin distance of 11–20 mm, and 22 (40%) patients had a margin distance >20 mm. The margin distance of patients in the segmentectomy group was comparable to the wedge resection group (17 ± 3.1 mm vs. 16 ± 4.2 mm; *P* = 0.235). In addition, we evaluated the oncological outcomes on the basis of operative procedure in patients in the MP positive and MP negative groups, and there was no difference in the MP negative group among wedge resection, segmentectomy or lobectomy on OS (*P* = 0.103) and RFS (*P* = 0.256) (Fig 3). However, in the MP positive groups, patients who underwent anatomic lobectomy or segmentectomy had a superior five‐year OS (*P* = 0.001) and RFS (*P* = 0.033) than those who underwent wedge resection. We also found that the sensitivity and specificity of frozen section diagnosis of presence of micropapillary component was 59.9% and 90.6%. The data of recurrence pattern of subcentimeter lung cancer with micropapillary component according to the surgical procedure is shown in Table 4. All recurrence (100%) in patients who underwent wedge resection was locoregional. Three quarters of recurrence (75%) in the anatomical resection group was locoregional. Locoregional recurrence was higher in patients who underwent wedge resection (Table 4).

**Table 4:** Patterns of recurrence in patients with subcentimeter lung adenocarcinoma with a micropapillary component based on surgical procedure

Characteristics	Anatomical resection (*N* = 85)	Wedge resection (*N* = 12)
Locoregional recurrence	13 (15)	5 (42)
Only hilar node	4 (5)	2 (17)
Only mediastinal node	4 (5)	1 (8)
Hilar and mediastinal node	1 (1)	1 (8)
Malignant effusion	2 (2)	0 (0)
Ipsilateral nodule	2 (2)	1 (8)
Distant recurrence	4 (5)	0 (0)
Adrenal gland	2 (2)	0 (0)
Contralateral nodule	2 (2)	0 (0)
Total	17 (20)	5 (42)

## Discussion

The new eighth edition of the tumor, node, and metastases (TNM) classification of NSCLC suggests that T component, especially the maximum tumor size, is the most important predictor of prognosis.^16^ T1 disease is subclassified into T1a (≤1 cm), T1b (> 1 to ≤2 cm) and T1c (>2 to ≤3 cm) diseases according to different survival outcomes in various tumor sizes.^17^ Considering the distinct prognosis of subcentimeter lung adenocarcinoma, in our study we aimed to evaluate the postoperative outcomes of this group of adenocarcinomas. As far as we are aware, our study is currently the first multicenter study to evaluate the prognostic significance of MP present in subcentimeter lung adenocarcinoma. Our study revealed that subcentimeter lung adenocarcinoma had an excellent prognosis, while patients with MP subtype are associated with a poor prognosis.

Previous studies have found that IASLC/ATS/ERS classification could predict prognosis for stage I lung adenocarcinoma.^18^, ^19^, ^20^ Yoshizawa *et al*. reported a five‐year RFS rates of lepidic predominant, papillary predominant, and acinar predominant subtypes were 90.0%, 83.0%, and 84.0% respectively, but only 70.0% and 67.0% in solid and MP predominant subtypes.^21^ However, previous studies have focused mainly on the relationship between prognosis and predominant histological patterns. Lung adenocarcinoma usually contains complex mixtures of different histological subtypes.^22^ A few studies have revealed that minor components of solid and/or MP subtypes are associated with nodal metastasis and poor prognosis.^23^, ^24^, ^25^, ^26^, ^27^ In our study, there were only four MP predominant cases, but MP components were found in 97 cases. As reported in a previous study,^23^ we found patients with lung adenocarcinomas harboring MP components had shorter RFS and OS.

In our study, solid‐predominant cases or solid present pattern cases did not show a shorter RFS or OS compared with other patterns which might be attributed to limited samples (five solid‐predominant and 14 solid present). However, we found solid present pattern cases of lung adenocarcinoma had significantly less heterogeneity with respect to the number of associated patterns when compared with others.

According to the current classification of lung adenocarcinoma, all adenocarcinoma subtypes are listed in 5% increments. However, there are studies which have revealed that even a small percentage of the MP pattern, such as <5% of the entire tumor, has a significant prognostic impact on OS.^23^ Thus, we should not only focus on MP predominant lung cancer cases, but also those harboring scant MP components.

Lobectomy and systematic mediastinal lymph node dissection as standard procedures of lung cancer surgery was established by the Lung Cancer Study Group about 20 years ago.^28^ However, some studies have reported that in well‐selected patients with small‐sized and/or early‐stage NSCLC, anatomic segmentectomy may achieve equivalently long‐term survival but preserve more lung parenchyma and pulmonary function than lobectomy.^29^ Previous studies have reported that segmentectomy, even wedge resection, might be adequate for early‐stage NSCLC such as adenocarcinoma in situ (AIS), minimally invasive adenocarcinoma (MIA), or invasive adenocarcinomas (IA) with lepidic predominant subtype. Our study also found that there was no significant difference between lobectomy and segmentectomy in subcentimeter lung adenocarcinoma with or without a MP component in RFS and OS. However, patients with subcentimeter lung adenocarcinoma with a MP component of 5% or greater treated with wedge resection are at a higher risk of recurrence than similar patients treated with anatomical resection. These consequences may be due to an insufficient surgical margin or limited lymph node dissection during wedge resection. Similarly, the OS and RFS of MP predominant subtypes were significantly worse than those in other predominant subtypes. Therefore, we recommend that segmentectomy or lobectomy with extensive lymph node dissection is necessary for MP subtype adenocarcinoma, although the lesions were subcentimeter nodules in our study.

There are some limitations in our study. First, this retrospective study was performed at seven medical centers but with a small sample size. Second, a selection bias was inevitable as procedures were performed by different surgeons. Third, the eighth TNM classification proposed the invasive component diameter instead of whole tumor size to be a superior choice of T staging measurement. Therefore, a prospective, multicenter, randomized, controlled study is warranted to validate the results.

In conclusion, patients with subcentimeter lung adenocarcinoma have a favorable prognosis. However, MP subtype is still an independent risk factor of worse survival in patients with subcentimeter lung adenocarcinoma. Thus, lobectomy or segmentectomy should be performed in patients with MP subtype despite tumor diameter ≤ 1 cm and a prospective study focusing on the diagnostic accuracy of MP by frozen section is necessary in the future.

## Disclosure

The authors have no conflicts of interest to declare.

## References

[1] Siegel RL , Miller KD , Jemal A . Cancer statistics, 2018. CA Cancer J Clin 2018; 68 (1): 7–30. 29313949 10.3322/caac.21442

[2] Travis WD , Brambilla E , Noguchi M *et al* International association for the study of lung cancer/american thoracic society/european respiratory society international multidisciplinary classification of lung adenocarcinoma. J Thorac Oncol 2011; 6 (2): 244–85. 21252716 10.1097/JTO.0b013e318206a221 PMC4513953

[3] Zhang Y , Wang R , Cai D *et al* A comprehensive investigation of molecular features and prognosis of lung adenocarcinoma with micropapillary component. J Thorac Oncol 2014; 9 (12): 1772–8. 25226429 10.1097/JTO.0000000000000341

[4] Ujiie H , Kadota K , Chaft JE *et al* Solid predominant histologic subtype in resected stage i lung adenocarcinoma is an independent predictor of early, extrathoracic, multisite recurrence and of poor postrecurrence survival. J Clin Oncol 2015; 33 (26): 2877–84. 26261257 10.1200/JCO.2015.60.9818 PMC4554749

[5] Cha MJ , Lee HY , Lee KS *et al* Micropapillary and solid subtypes of invasive lung adenocarcinoma: Clinical predictors of histopathology and outcome. J Thorac Cardiovasc Surg 2014; 147 (3): 921–928 e922. 24199757 10.1016/j.jtcvs.2013.09.045

[6] Yuan Y , Ma G , Zhang Y , Chen H . Presence of micropapillary and solid patterns are associated with nodal upstaging and unfavorable prognosis among patient with ct1n0m0 lung adenocarcinoma: A large‐scale analysis. J Cancer Res Clin Oncol 2018; 144 (4): 743–9. 29392402 10.1007/s00432-017-2571-7 PMC11813456

[7] National Lung Screening Trial Research T , Aberle DR , Adams AM *et al* Reduced lung‐cancer mortality with low‐dose computed tomographic screening. N Engl J Med 2011; 365 (5): 395–409. 21714641 10.1056/NEJMoa1102873 PMC4356534

[8] Yu X , Li Y , Shi C , Han B . Risk factors of lymph node metastasis in patients with non‐small cell lung cancer </= 2 cm in size: A monocentric population‐based analysis. Thorac Cancer 2018; 9 (1): 3–9. 29034994 10.1111/1759-7714.12490 PMC5754297

[9] Chen B , Wang X , Yu X *et al* Lymph node metastasis in Chinese patients with clinical t1 non‐small cell lung cancer: A multicenter real‐world observational study. Thorac Cancer 2019; 10 (3): 533–42. 30666800 10.1111/1759-7714.12970 PMC6397906

[10] Altorki NK , Wang X , Wigle D *et al* Perioperative mortality and morbidity after sublobar versus lobar resection for early‐stage non‐small‐cell lung cancer: Post‐hoc analysis of an international, randomised, phase 3 trial (calgb/alliance 140503). Lancet Respir Med 2018; 6 (12): 915–24. 30442588 10.1016/S2213-2600(18)30411-9 PMC6396275

[11] Gu C , Wang R , Pan X *et al* Sublobar resection versus lobectomy in patients aged </=35 years with stage ia non‐small cell lung cancer: A seer database analysis. J Cancer Res Clin Oncol 2017; 143 (11): 2375–82. 28819775 10.1007/s00432-017-2499-y PMC11819410

[12] Tsutani Y , Tsubokawa N , Ito M *et al* Postoperative complications and prognosis after lobar resection versus sublobar resection in elderly patients with clinical stage i non‐small‐cell lung cancer. Eur J Cardiothorac Surg 2018; 53 (2): 366–71. 28958068 10.1093/ejcts/ezx296

[13] National comprehensive cancer network . Nccn clinical practice guidelines in oncology (nccn guidelines): Non‐small cell lung cancer, version 5. 2019. [Cited 6/11/2018] Available from URL: https://www.nccn.org/patients/guidelines/cancers.aspx#nsclc.

[14] Nitadori J , Bograd AJ , Kadota K *et al* Impact of micropapillary histologic subtype in selecting limited resection vs lobectomy for lung adenocarcinoma of 2 cm or smaller. J Natl Cancer Inst 2013; 105 (16): 1212–20. 23926067 10.1093/jnci/djt166 PMC3748005

[15] Travis WD , Brambilla E , Nicholson AG *et al* The 2015 world health organization classification of lung tumors: Impact of genetic, clinical and radiologic advances since the 2004 classification. J Thorac Oncol 2015; 10 (9): 1243–60. 26291008 10.1097/JTO.0000000000000630

[16] Goldstraw P , Chansky K , Crowley J *et al* The iaslc lung cancer staging project: Proposals for revision of the tnm stage groupings in the forthcoming (eighth) edition of the tnm classification for lung cancer. J Thorac Oncol 2016; 11 (1): 39–51. 26762738 10.1016/j.jtho.2015.09.009

[17] Rami‐Porta R , Bolejack V , Crowley J *et al* The iaslc lung cancer staging project: Proposals for the revisions of the t descriptors in the forthcoming eighth edition of the tnm classification for lung cancer. J Thorac Oncol 2015; 10 (7): 990–1003. 26134221 10.1097/JTO.0000000000000559

[18] Takahashi Y , Eguchi T , Kameda K *et al* Histologic subtyping in pathologic stage i‐iia lung adenocarcinoma provides risk‐based stratification for surveillance. Oncotarget 2018; 9 (87): 35742–51. 30515266 10.18632/oncotarget.26285 PMC6254662

[19] Tsao MS , Marguet S , Le Teuff G *et al* Subtype classification of lung adenocarcinoma predicts benefit from adjuvant chemotherapy in patients undergoing complete resection. J Clin Oncol 2015; 33 (30): 3439–46. 25918286 10.1200/JCO.2014.58.8335 PMC4606061

[20] Hung JJ , Yeh YC , Jeng WJ *et al* Predictive value of the international association for the study of lung cancer/american thoracic society/european respiratory society classification of lung adenocarcinoma in tumor recurrence and patient survival. J Clin Oncol 2014; 32 (22): 2357–64. 24799473 10.1200/JCO.2013.50.1049

[21] Yoshizawa A , Motoi N , Riely GJ *et al* Impact of proposed iaslc/ats/ers classification of lung adenocarcinoma: Prognostic subgroups and implications for further revision of staging based on analysis of 514 stage i cases. Mod Pathol 2011; 24 (5): 653–64. 21252858 10.1038/modpathol.2010.232

[22] Travis WD , Brambilla E , Burke AP , Marx A , Nicholson AG . Introduction to the 2015 world health organization classification of tumors of the lung, pleura, thymus, and heart. J Thorac Oncol 2015; 10 (9): 1240–2. 26291007 10.1097/JTO.0000000000000663

[23] Zhao Y , Wang R , Shen X *et al* Minor components of micropapillary and solid subtypes in lung adenocarcinoma are predictors of lymph node metastasis and poor prognosis. Ann Surg Oncol 2016; 23 (6): 2099–105. 26842488 10.1245/s10434-015-5043-9 PMC4858562

[24] Lee G , Lee HY , Jeong JY *et al* Clinical impact of minimal micropapillary pattern in invasive lung adenocarcinoma: Prognostic significance and survival outcomes. Am J Surg Pathol 2015; 39 (5): 660–6. 25724001 10.1097/PAS.0000000000000399

[25] Suzuki M , Yokose T , Nakayama H . Prognostic contribution of non‐predominant solid and micropapillary components in lung adenocarcinomas. J Thorac Dis 2017; 9 (3): 504–6. 28449456 10.21037/jtd.2017.03.15 PMC5394081

[26] Wang Y , Zheng D , Zheng J *et al* Predictors of recurrence and survival of pathological t1n0m0 invasive adenocarcinoma following lobectomy. J Cancer Res Clin Oncol 2018; 144 (6): 1015–23. 29532227 10.1007/s00432-018-2622-8 PMC11813495

[27] Dai C , Xie H , Kadeer X *et al* Relationship of lymph node micrometastasis and micropapillary component and their joint influence on prognosis of patients with stage i lung adenocarcinoma. Am J Surg Pathol 2017; 41 (9): 1212–20. 28692600 10.1097/PAS.0000000000000901

[28] Ginsberg RJ , Rubinstein LV . Randomized trial of lobectomy versus limited resection for t1 n0 non‐small cell lung cancer. Lung cancer study group. Ann Thorac Surg 1995; 60 (3): 615–22 discussion 622‐613. 7677489 10.1016/0003-4975(95)00537-u

[29] Liu S , Wang R , Zhang Y *et al* Precise diagnosis of intraoperative frozen section is an effective method to guide resection strategy for peripheral small‐sized lung adenocarcinoma. J Clin Oncol 2016; 34 (4): 307–13. 26598742 10.1200/jco.2015.63.4907

